# Concurrent Validity of a Novel Wireless Inertial Measurement System for Assessing Trunk Impairment in People with Stroke

**DOI:** 10.3390/s20061699

**Published:** 2020-03-18

**Authors:** Norah Alhwoaimel, Martin Warner, Ann-Marie Hughes, Federico Ferrari, Jane Burridge, Seng Kwee Wee, Geert Verheyden, Ruth Turk

**Affiliations:** 1 School of Health Sciences, University of Southampton, Southampton SO17 1BJ, UK; m.warner@soton.ac.uk (M.W.); A.Hughes@soton.ac.uk (A.-M.H.); J.H.Burridge@soton.ac.uk (J.B.); R.Turk@soton.ac.uk (R.T.); 2 Department of Physical Therapy and Rehabilitation, Prince Sattam Bin Abdulaziz University, Alkharj 11942, Saudi Arabia; 3 Department of Neurosciences, Biomedecine and Movement Sciences, University of Verona, 37134 Verona, Italy; federico.ferrari_01@studenti.univr.it; 4 Department of Rehabilitation, Sacro Cuore Don Calabria Hospital, 37024 Negrar, Italy; 5 Centre for Advanced Rehabilitation Therapeutics (CART), Tan Tock Seng Hospital, Singapore 308433, Singapore; SengKwee.Wee@Singaporetech.edu.sg; 6 Health and Social Sciences Cluster, Singapore Institute of Technology, Singapore 138683, Singapore; 7 Department of Rehabilitation Sciences, KU Leuven—University of Leuven, 3001 Leuven, Belgium; geert.verheyden@kuleuven.be

**Keywords:** objective assessment, inertial sensor, trunk impairment scale, instrumented trunk impairment scale, stroke, validity

## Abstract

*Background:* The Trunk Impairment Scale (TIS) is recommended for clinical research use to assess trunk impairment post-stroke. However, it is observer-dependent and neglects the quality of trunk movements. This study proposes an instrumented TIS (iTIS) using the Valedo system, comprising portable inertial sensors, as an objective measure of trunk impairment post-stroke. *Objective*: This study investigates the concurrent and discriminant ability of the iTIS in chronic stroke participants. *Methods:* Forty participants (20 with chronic stroke, 20 healthy, age-matched) were assessed using the TIS and iTIS simultaneously. A Spearman rank correlation coefficient was used to examine concurrent validity. A ROC curve was used to determine whether the iTIS could distinguish between stroke participants with and without trunk impairment. *Results:* A moderate relationship was found between the observed iTIS parameters and the clinical scores, supporting the concurrent validity of the iTIS. The small sample size meant definitive conclusions could not be drawn about the parameter differences between stroke groups (participants scoring zero and one on the clinical TIS) and the parameter cut-off points. *Conclusions:* The iTIS can detect small changes in trunk ROM that cannot be observed clinically. The iTIS has important implications for objective assessments of trunk impairment in clinical practice.

## 1. Introduction

Trunk control is a vital part of balance and postural control; it plays an important role in holding the body upright and in performing selective trunk movements during static and dynamic postural adjustments [1,2]. Impairment of trunk control due to trunk muscle weakness, poor control or reduced position sense results in decreased balance and an increased risk of falls; it also interferes with the performance of daily living activities, such as turning in bed, sitting, rising from sitting to standing and walking [3,4,5]. Moreover, stability of the upper trunk is considered to be a prerequisite of upper limb function and hand usage [6]. The role of trunk ability is often overlooked as an integral part of the recovery process; trunk control has been recognized to be an important early predictor of functional recovery after stroke explaining 45% to 71% of the variance of functional recovery post-stroke [7,8].

In stroke, impaired trunk control can be assessed using clinical outcome measures such as the Trunk Control Test (TCT) and the Trunk Impairment Scale (TIS) [9,10]. The TIS is recommended for use in clinical research because it has sufficient psychometric properties demonstrated in a high concurrent validity (r = 0.83), excellent test–retest reliability (ICC = 0.96), excellent inter-rater reliability (ICC = 0.99) and has no ceiling effect [10,11,12]. The TIS consists of three sub-sections which assess static sitting balance, dynamic sitting balance and trunk coordination. Following Rasch analysis the static sitting balance subscale was eliminated, and the scale was renamed TIS Version 2.0 (TIS-V2) [13]. However, although the TIS is commonly used to evaluate trunk impairment post-stroke, a limitation is that the scale uses ordinal scores to measure trunk impairment by means of the degree of task completion without considering movement quality during task performance. More detailed information on trunk movement quality can obtained by kinematic analysis of several indices (i.e., speed, smoothness and range of movement). Identifying these stroke-related impairment kinematic characteristics potentially offers a better understanding of the relationship between movement quality and performance during neurorehabilitation and stroke recovery. A recent study which measured trunk movement using 3D kinematic analysis found that the lateral pelvic ROM was the best predictor (R^2^ = 0.2 and *p <* 0.006) of clinical recovery measured with the TCT [14]. It is critical to differentiate between compensatory and non-compensatory movement after neurorehabilitation and kinematic measures may help to identify deficits even in people who have recovered well from their stroke [15]. The majority of published literature focuses on measuring trunk movement in relation to upper limb recovery, and there is a dearth of evidence regarding recommended kinematic parameters to use for evaluating trunk impairment [14,16]. An instrumented version of TIS (iTIS) to address this limitation is therefore warranted. 

Optoelectronic measurement systems can be used to quantify trunk movements in research settings [17]. However, these systems are not readily clinically available because they entail a high cost and require a large installation space. To overcome these limitations, the use of alternative, objective low-cost measuring systems such as inertial measurement units (IMUs) may be useful. The Valedo system (Hocoma, Switzerland) is a wireless movement analysis system that comprises three lightweight sensors (IMUs) that measure trunk movement (in degrees) and the velocity of body segments with respect to magnetic fields and gravity in a non-invasive way [18,19]. Recent research has examined the concurrent validity and reliability of the Valedo system using an optoelectronic system as the gold standard for measuring 3D trunk movement in healthy participants and found that the Valedo system is a valid (r^2^ coefficients > 0.94) and reliable (3%–9% coefficient of variation) measure for estimating trunk movement [18]. The instrumentation of the TIS could potentially provide more detailed and clinically relevant information about trunk movement and it’s relation to trunk impairment for stroke participants. To our knowledge, this is the first study that has instrumented a clinical trunk impairment scale. It was therefore the aim of this cross-sectional observational study to instrument the TIS using the Valedo system (iTIS) and correlate it to the gold-standard TIS (cTIS-V2) for concurrent validity testing. This will additionally establish the discriminant ability of the iTIS, as the scores of chronic stroke subjects and healthy, age-matched subjects can be compared. We hypothesize that the iTIS is a valid tool for the evaluation of trunk impairment in chronic strokes and is able to discriminate between people who do, and those do not have trunk impairment using the cTIS-V2 as a gold standard.

## 2. Materials and Methods

(1) Participants

Forty participants (20 with chronic stroke and 20 healthy, age-matched) were recruited from the University of Southampton (School of Health Sciences’ Research Participant Register), the Hobbs Rehabilitation Centre and local stroke clubs (Hampshire, UK). The inclusion criteria for healthy participants were that the individuals were between 40 and 80 years old and able to understand and follow simple instructions. In addition, the stroke participants needed to have trunk impairment resulting from the stroke (cTIS ≤ 20) and the ability to maintain a seated position for 10 s. The exclusion criteria for both groups were acute low back pain, a history of spontaneous fractures, a hip prosthesis, uncontrolled epileptic seizures, implanted ferromagnetic materials or active devices within the body, and skin diseases or lesions near the sensor placement. After potential participants demonstrated an interest in taking part in the study, a screening telephone call was made by the researcher to ensure that the participant matched the inclusion and exclusion criteria. All participants were encouraged to wear vest tops and comfortable trousers to enable the researchers to attach the sensors to the participants’ skin easily. The Ethics Committee of University of Southampton approved this study (ethical approval code: 25280) and all participants signed informed consent.

(2) Measurement Protocol 

**Figure 1:**
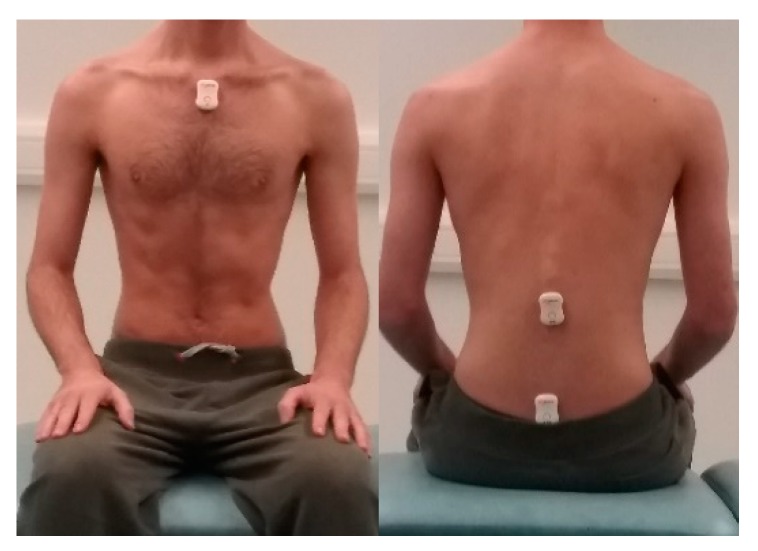
Sensor placements.

While the participant was standing, three lightweight Valedo sensors (Hocoma, Switzerland) were placed using double-sided sticky tape: sensor one on the sacral spinal level S1, sensor two on the L1 spinal level and sensor three on the sternum (Figure 1). To mitigate measurement error by ensuring the same placement of the sensors was made by assessors, the following anatomical body landmarks were used to identify the S1 and L1 levels: the anterior superior iliac spine (ASIS) along the iliac crest to L4 and then palpation upward and downward to locate L1 and S1 respective, whilst the third sensor was placed 2.5 cm below the sternal notch. Training on how to place the sensors was provided and standardized by the assessors (and could potentially be given to anyone). The sensors were placed and removed by the same assessor. The Valedo sensors can record a ±0.1° range of motion over a range of 360° around all axes (Valedo User Manual, Hocoma). The recorded data output, formatted as an Excel file, indicates the rotation of each sensor at X, Y and Z direction on the three body planes (sagittal, frontal and transverse) over the duration of the task.

The participant was then seated on a plinth without any back support, their hip and knees were flexed at 90°, and they were barefoot with their feet resting on the floor. The participants were asked to perform the 14 cTIS-V2 tasks. Each task was demonstrated by the assessor to the participant before they performed it. Every participant was assessed using the clinical and instrumented versions of the TIS-V2 simultaneously.

(3) Development of the instrumented Trunk Impairment Scale (iTIS) 

In previous literature, no consensus exists on the best kinematic parameters to be used for in the evaluation of trunk movement [14]. Therefore, the parameters of interest for each TIS task were determined based on the clinical reasoning of the team, taking into account the maximum range of movement in each direction expected from performance of the task and direction of movement by the author and research team (Turk, Warner, Hughes, and Ferrari). Initially the following parameters were exported for each dynamic task: flexion, extension and lateral flexion on both sides for the sternal and sacral sensors. Following completion of the data collection, the data were analyzed to identify the most appropriate and important kinematic parameters to be reported in each task. For the dynamic subscale parameters, the degree of range of motion (ROM) of lateral flexion to either the affected or unaffected side was considered. For the coordination subscale, the degree of lumbar and sternal ROM towards both sides were measured, and the symmetry of rotation movement between the affected and unaffected sides was considered. The symmetry between both sides was calculated as a percentage (%) (i.e., 100% symmetry means that the rotation ROM on both sides is equal). The parameters of interest are presented in Table 1. The data exported as an excel file for each task performed. Then, all of the recorded tasks processed by MATLAB (MATLAB R2016a) (The MathWorks, Inc.) to extract the parameters used for iTIS. The MATLAB script The MATLAB algorithms were written by an experienced musculoskeletal biomechanics researcher and performed by the author N.A. The MATLAB scripts for the data processing from raw data to ROM is documented in Supplementary Materials.

(4) Statistical Analysis

The data were imported into Excel and analyzed using IBM SPSS Statistics 24 (SPSS Inc., Chicago, IL, USA). Descriptive statistics were used to summarize the demographic data and the parameters of interest. The normality of the data was checked using the Shapiro–Wilks test.

To evaluate the concurrent validity of the Valedo system in measuring trunk impairment, the correlation between clinical scores (using TIS-V2) and instrumental scores (using iTIS) was examined using a Spearman correlation coefficient analysis [20]. The correlation coefficient ranges from −1 to +1 to reflect the strength of the relationship between the variables. The positive or negative sign of the coefficient indicates positive or negative correlation [20]. The following correlation classification was used to interpret the correlation coefficient result: none or very low: *ρ* = 0–0.25; low: *ρ* = 0.26–0.40; moderate: *ρ* = 0.41–0.69; high: *ρ* = 0.70–0.89; very high: *ρ* = 0.90–1.0 [21]. Following this, for the coordination subscale a conservative adjustment to compensate for the number of similar tasks (task 1 and 2, task 3 and 4) with four parameters of interest for each task was applied to achieve a family wise significance Bonferroni level of 5% (test-wise significant levels 0.6%).

The score differences of the stroke participants and the healthy, age-matched participants were assessed using an independent samples *t*-test. In addition, the difference between the participants with stroke who achieved scores of one or two and those who scored zero on the cTIS-V2 tasks was calculated using an independent samples *t*-test and a one-way ANOVA. 

A receiver operating characteristic (ROC) curve analysis and the area under curves (AUCs) were used to determine the cut-off point of the iTIS parameters for distinguishing trunk impairment (i.e., participants who scored zero, one and two on the cTIS-V2) in the stroke group [22]. The cut-off point was determined using the Youden index (Youden index = sensitive value + specificity value − 1) at the point where both the sensitivity and specificity values were maximized [23]. In this study, the cut-off point will be the best representative point of the degree of trunk ROM recorded by the Valedo sensors that can distinguish between stroke participants with trunk impairment (scored zero on cTIS tasks) and those without trunk impairment (scored one, or two on cTIS tasks). The area under curve (AUC) is a summary measure of the accuracy of a quantitative diagnostic test. The maximum AUC is the best cut-off score. The AUC values were interpreted according to an arbitrary guideline; one could distinguish between no (AUC < 0.5), poor (0.5 ≤ AUC < 0.7), acceptable (0.7 ≤ AUC < 0.8), excellent (0.8 ≤ AUC < 0.9) and outstanding (AUC > 0.9) discriminant ability. 

## 3. Results 

### 3.1. Participant Characteristics

Twenty adults with chronic stroke and resulting trunk impairment (mild to severe) and 20 healthy, aged-matched controls were recruited. The participants’ characteristics are presented in Table 2.

### 3.2. Concurrent Validity 

Significant moderate correlations (negative) were observed between the cTIS-V2 score and iTIS parameters of the dynamic subscale: lateral flexion to affected side in Tasks 1 and 2 (r = −0.59, *p* < 0.006) and lateral flexion to the unaffected side in Tasks 4 and 5 (r = −0.52, *p* < 0.02) (Table 3). Furthermore, significant moderate correlations were observed between the cTIS-V2 score and lateral flexion to the affected side in Task 7 (r = 0.52, *p* < 0.01) and lateral flexion to the unaffected side in Task 9 (r = 0.47, *p* < 0.03). The remaining parameters for Tasks 3, 6, 8 and 10 in the dynamic subscale, which all assessed compensatory movement, resulted in a very low, non-significant correlation (r ≤ 0.26). 

For the coordination subscale, significant high correlations were observed between the cTIS-V2 score and two variables, including symmetry in Task 2 (rotate upper trunk 6 times within 6 s) (r = 0.71, *p* < 0.001) and the total number of rotations in Task 3 (rotate lower trunk 6 times) (r = 0.73, *p* < 0.001) (Table 4). Furthermore, significant moderate correlations were shown between the cTIS-V2 scores and the following iTIS parameters: symmetry in Task 1 (rotate upper trunk 6 times); total number of rotations in Tasks 1, 2 and 3; and rotation to the affected side in Task 1 (r ≤ 0.64). Following a conservative Bonferroni adjustment three associations were statistically significant at *p* ≤ 0.006, namely symmetry in Tasks 2 and 4, and the total number of rotations in Task 2. The rotation to the unaffected side recorded low and very low correlations using the cTIS-V2 scores (r ≤ 0.34) in all tasks.

### 3.3. Differences between Groups

Difference between stroke participants and healthy, age-matched participants: the differences in trunk lateral flexion between stroke participants and healthy participants were significantly different (*p*-value range: 0.001 to 0.05) in seven tasks of the dynamic subscale (Tasks 1, 2, 3, 6, 8, 9 and 10) (Table 5). The remaining three tasks (Tasks 4, 5 and 7) showed a non-significant difference between the groups (*p* > 0.05). In the coordination subscale, the average rotations to both the affected and unaffected sides were significantly different between stroke and healthy participants for all tasks (*p*-value range: 0.001 to 0.05). However, the symmetry parameter indicated a non-significant difference between the groups for all tasks (*p* > 0.05). For the last parameter, the total number of rotations, there was a significant difference between the stroke and healthy participants for only two tasks (Tasks 2 and 4; *p*-value < 0.05).

(1) Difference between stroke participants who scored two and one on the cTIS and stroke participants who scored zero

In the dynamic subscale, the stroke participants who scored one showed a lower trunk ROM during Tasks 1–6 and a higher trunk ROM during Tasks 7–10 compared to stroke participants who scored zero, as presented in Table 6. The differences in trunk lateral flexion between stroke participants who scored one and those scoring zero were significantly different (*p* ≤ 0.05) for four tasks (Tasks 1, 4, 7 and 9). The remaining tasks (Tasks 3, 6, 8 and 10) showed a non-significant difference between groups (*p* > 0.05). In the coordination subscale, the average rotation to both the affected and unaffected sides showed a non-significant difference between groups for all tasks (*p* > 0.05) except for Task 1. In Task 1, only the average rotation to the unaffected side was significantly different between groups (*p* = 0.002). In contrast, the total number of rotations parameter showed a high significant difference between the groups for all tasks (*p*-value range: 0.05 to 0.001). For the symmetry parameter, there was a significant difference between stroke groups for only two tasks (Tasks 1 and 2; *p*-value < 0.05). 

### 3.4. ROC Curve Analysis

Table 7 summarizes the results of the ROC curve analysis, including the AUCs and the identified cut-off score for each iTIS parameter that discriminated between stroke participants who scored zero, one and two for each task, along with their sensitivity and specificity. 

**Figure 2:**
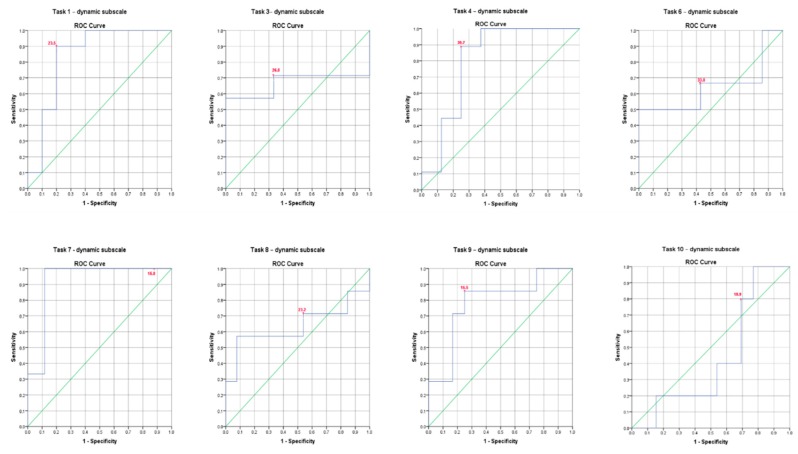
Receiver operator curves (ROC) of the iTIS dynamic subscale parameters to distinguish between stroke participants with impairment (scored zero on cTIS-V2 tasks) and those without trunk impairment (scored one, or two on cTIS-V2 tasks). * indicates cut-off points.

In the dynamic subscale tasks, one parameter (lateral flexion to the affected side in Task 7) was considered a highly accurate test (AUC = 0.92), indicating outstanding discriminant ability to distinguish between people who scored zero and who scored one on the cTIS-V2 tasks (Figure 2). Three out of eight parameters (lateral flexion to affected side in Task 1, lateral flexion to the unaffected side in both Tasks 4 and 9) showed an AUC range of 0.78–0.84, indicating acceptable to excellent discriminant ability. The remaining four parameters were less accurate, with an AUC range between 0.43 to 0.67, indicating no or poor discriminant ability.

**Figure 3:**
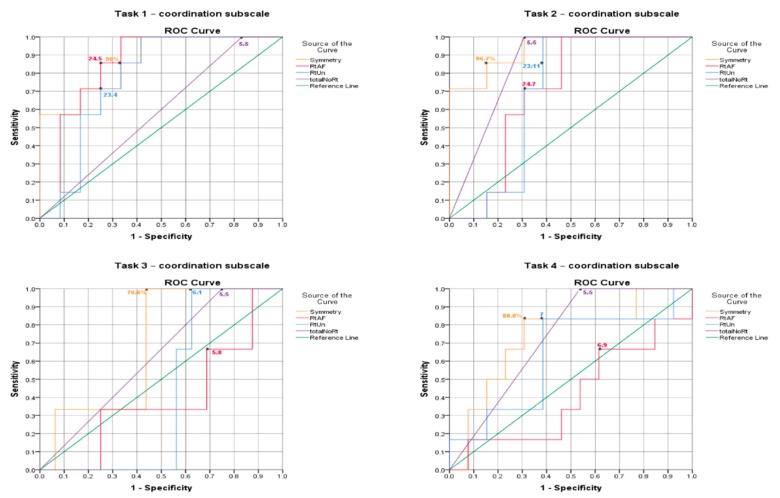
Receiver operator curves (ROC) of the iTIS coordination subscale parameters to distinguish between stroke participants with impairment (scored zero on cTIS-V2 tasks) and those without trunk impairment (scored one, or two on cTIS-V2 tasks). * indicates cut-off points.

In the coordination subscale, the symmetry parameter in Task 2 demonstrated an outstanding discriminant ability (AUC = 0.93), and acceptable to excellent discriminative ability (AUC = 0.70–0.87) in the remaining tasks (Tasks 1, 3 and 4) (Figure 3). For the average rotation to the affected side parameter, the AUC for Tasks 1 and 2 (0.85 and 0.70, respectively) indicated excellent and acceptable discriminative ability. In contrast, the average rotations to the affected side parameter in Tasks 3 and 4 had no discriminative ability (AUC = 0.40 and 0.41, respectively). For the average rotation to the unaffected side parameter, the AUC in Tasks 1 and 2 (0.77 and 0.70, respectively) showed acceptable discriminative ability, while Tasks 3 and 4 were found to have no discriminative ability. The last parameter was the total number of rotations, which was found to have poor discriminative ability in Tasks 2 and 4 (AUC ≤ 0.63) and acceptable to excellent discriminative ability in Tasks 1 and 3 (AUC ≥ 0.73).

## 4. Discussion

The present study demonstrated a moderate relationship between most of the observed iTIS parameters and the clinical score of the cTIS-V2, supporting the concurrent validity of the instrumented TIS using the Valedo system. A few parameters demonstrated a weak relationship between the iTIS and the clinical score of the cTIS-V2, including trunk lateral flexion in Task 3, 6, 8 and 10 in the dynamic subscale and rotation towards the unaffected side in all tasks of the coordination subscale.

### 4.1. Validity 

The validity of the iTIS was demonstrated by correlating the iTIS parameters to the clinical scores of the cTIS-V2 using a Spearman rank correlation. The results indicated a moderate negative correlation in the dynamic subscale parameters for Tasks 1, 2, 4 and 5 (touching the bed with the affected and unaffected elbow). The negative correlation arose because of the higher lateral flexion ROM towards the affected and unaffected sides recorded by stroke participants who scored zero compared to those who scored one on the clinical TIS. This was because most of the participants (n = 12/20) lost their sitting balance and fell to their side. This is in line with the findings of Jijimol et al. (2013), who found a significant high correlation (r = 0.91, *p* < 0.01) between balance and TIS [24]. Only one participant who scored zero did not lose her balance; she had a low ROM because she was not able to touch the bed with her elbow due to lacking in ROM and stopped moving during her performance of Tasks 4 and 5.

The correlation in Tasks 7 and 9 (lift pelvis from bed on the affected and unaffected side) of the dynamic subscale had moderate positive correlations. The results could be explained by the higher lateral flexion ROM values reported in people who scored one compared to those who scored zero on the clinical TIS. Moreover, for the clinical TIS in Task 7, only two stroke participants scored zero, while 18 participants scored one. In Tasks 3, 6 and 8 in the dynamic subscale, poor correlation values between the clinical and instrumented TIS can be explained by the presence of compensatory movements (e.g., the use of the upper limb, the contralateral hip abduction, the hip flexion, the knee flexion, sliding of the feet and the loss of contact between the heel and the floor), which if observed by the assessor, in the clinical TIS (dynamic subscale), a score of zero is given. However these could not be measured by the setup of the Valedo sensors, as the sensors were only attached to the trunk. This compensatory movement can be measured with an alternative method of application of the sensors (i.e., fixing one of the sensors on the lower limb) to be able to record the movement. The compensation movements observed are likely to be due to impaired postural control and weight-shifting ability during the performance of these tasks. The findings from this study are consistent with those of Messier et al. (2004), who reported a significantly lower weight-bearing ability in chronic stroke participants for both paretic and non-paretic feet when comparing a weight-bearing value on both the dominant and non-dominant feet in healthy, age-matched participants (*p* = 0.05) during the performance of trunk lateral flexion (45 degrees) from a sitting position [25]. Furthermore, lower limb sensory deficits may have affected the trunk movements; it has been suggested that somatosensory information from the feet determines how people with stroke adjust themselves on a support surface [26]. Another possible cause of the reduction in ROM for those who scored zero in the pelvis-lifting tasks may result from the reduction in the activity of the rectus abdominis and latissimus dorsi muscles on the affected side of the body in comparison to the unaffected side.

For the coordination subscale, the symmetry showed a moderate to high correlation of iTIS to cTIS for Tasks 1 and 2 (rotate upper trunk 6 times), while for Tasks 3 and 4 (rotate lower trunk 6 times), the correlation was low. The result could be explained by the low values of lower trunk rotation ROM detected by the sacral sensor in Tasks 3 and 4, whereas in the clinical scale rotation of the lower trunk is easily observable through forward and backward movements of the knees. As a result of the low ROM values, a small change in rotation leads to asymmetry. In addition, a combination of factors such as spasticity in the lower extremities, weakness of the trunk and proximal lower extremity muscles can contribute to the difficulty performing rotation of the pelvis [2,25]. This explanation is supported by Verheyden et al. (2005), suggesting that identifying this movement in stroke participants is more difficult [12]. Furthermore, the total number of rotations demonstrates a moderate to high correlation, as stroke participants with a moderately impaired trunk were unable to complete six rotation movements within six seconds. Rotation ROM towards the affected and unaffected side in general showed a low correlation with the cTIS. This parameter is not measured in the cTIS (whereas symmetry and total number of rotations are), so whilst it may provide additional information about the quality of movement, the results suggest this parameter should be excluded from the iTIS because it does not contribute to the validity. Therefore the important parameters to be considered in the co-ordination subscale of iTIS should be symmetry and total number of rotations This is supported by the results of the Bonferroni adjustment which showed significant correlation at *p* ≤ 0.006 was noted in symmetry (Tasks 2 and 3) and total number of rotations (Task 2) parameters. 

### 4.2. Differences between Groups

The results of the *t*-test and the ROC curve analysis indicated the ability of the iTIS dynamic subscale parameters to distinguish both between participants with and without stroke as well as those stroke participants with and without trunk impairment. As expected, the performance of the stroke participants was lower than that of the healthy controls in most of the dynamic subscale tasks. This may be explained by the difficulties stroke participants face in fine-tuning the length of the lateral trunk muscles according to the task requirement compared to the healthy controls [27]. The non-significant difference in some of the dynamic subscale tasks (Tasks 3, 6, 8 and 10) between stroke participants who scored zero and those who scored one on the clinical TIS-V2 is likely to be due to the unmeasured compensatory movements mentioned previously. The participants who scored one on the cTIS-V2 recorded a high trunk lateral flexion ROM in the dynamic subscale tasks, while those who scored zero reached a high ROM by compensating (using the upper or lower limb), which explains the non-significant difference in some of the dynamic subscale tasks.

In the coordination subscale, the differences between the stroke participants and the healthy group were significantly different in their amount of axial rotation ROM in the direction of both the affected and unaffected sides, suggesting a decrease in trunk rotational ability in the stroke group. This finding is supported by (Tanaka et al. 1997), who found a significant reduction in trunk rotatory muscle performance in stroke participants compared to healthy, age-matched participants during trunk rotation at angular velocities of 60, 120, and 150 degrees per second (*p* < 0.05) [28]. However, the symmetry parameters were not significantly different between the groups, and this could be because of the compensatory strategy used by stroke participants to perform coordination subscale tasks. Some of the stroke participants completed the tasks with compensation, resulting in a symmetrical movement during the performance of the coordination tasks (e.g., the participants leaned to both sides instead of rotating the lower trunk in Tasks 3 and 4 or carried the affected arm with the other hand to assist the movement of the upper trunk rotation in Tasks 1 and 2). In contrast, the number of rotations shows a significant difference between healthy and stroke participants in Tasks 2 and 4, when the task was required to be completed within six seconds. This could be explained by a reduction in the participants’ ability to initiate the trunk movement in the stroke group; onset latencies of trunk muscles (lumbar erector spinae) have been shown to be delayed in stroke participants when compared with healthy, age-matched controls (*p* < 0.04) [27].

### 4.3. Limitations 

This study had several limitations. The methodology limitation which affected the validity results was that the three sensors used in this study were placed solely on the trunk, so were unable to detect any compensation by the upper or lower limbs during the task performance. 

System errors may also have affected both the validity and reliability [29]. The Valedo system crashed during the completion of the tasks during some sessions. Although the data were deleted, the task was performed again and the next data recording was checked, it is possible that potential errors in the system’s records from before the crash could have affected the recording. The system crashes during the test performance may have been due to using the Valedo system for more than 6 h continuously recording data leading to a slower laptop performance. When we removed the recorded data to another hard drive, the problem was solved. 

The sample size was too small to allow definitive conclusions to be drawn about the differences in parameters between stroke groups (stroke participants who scored zero and one on the clinical TIS) or to establish cut-off points for each parameter. All stroke data were used to perform the cut-off point analysis. This might have led to the performance of the proposed approach being overestimated, especially because of the small number of participants. In addition, due to the nature of clinical TIS scoring, which assumes that people who score zero on Tasks 1 and 2 will automatically score zero in Task 3, fewer than 20 participants performed Task 3. The findings indicate that a higher number of stroke participants are needed to robustly investigate the validity and reliability of the iTIS.

## 5. Clinical Implications and Recommendations

Clinicians commonly use clinical tests such as the TIS to assess trunk impairment and to monitor changes in impairment after intervention. This paper presents the iTIS as an objective tool to enable the clinician to assess and monitor the trunk impairment considering the quality of the trunk movement. It will give them detailed information about trunk movement by quantifying the trunk ROM in each task. However, the iTIS exhibits a limitation in detecting the compensatory movement exerted by lower limb which is commonly observed clinically. To overcome this limitation, a further research study to investigate the ability of Valedo system to detect compensatory movements during performance of the iTIS by moving one of the trunk sensors to the lower limb is warranted.

## 6. Conclusions

This study is the first to demonstrate the feasibility of an iTIS. Moderate validity has been shown, and different methods of application of the iTIS could be used where the validity was low. Future studies with larger sample sizes and a robust standardized application of iTIS using additional sensors on the upper and/or lower limbs to detect compensation are warranted to establish reference data for iTIS parameters.

The iTIS provides more information about trunk performance than the cTIS-V2, as it has the ability to detect small changes in trunk ROM that may not be observed clinically. These findings indicate that the inertial sensor-based iTIS measures have important implications for the objective assessment of trunk impairment in clinical practice. The data-rich set could be useful in the customization of physiotherapy to address individuals’ trunk control issues. Another potential use of the iTIS is to advance research into understanding the mechanisms of trunk control and compensatory trunk movements throughout recovery from a stroke.

## Figures and Tables

**Table 1:** Sensor location, plane of movement and parameters of interest for each TIS-V2 task.

cTIS-V2 Tasks	Sensor	Parameter of Interest	Plane of Movement
**Dynamic Sitting Balance Subscale**			
1—Touch the bed with the hemiplegic elbow	Sternum	ROM of lateral flexion to non-dominant/affected * side (degrees)	Frontal
2—Repeat Item 1	Sternum	ROM of lateral flexion to non-dominant/affected side (degrees)	Frontal
3—Repeat Item 1	Sternum	ROM of lateral flexion to non-dominant/affected side (degrees)	Frontal
4—Touch the bed with the unaffected elbow	Sternum	ROM of lateral flexion to dominant/unaffected side (degrees)	Frontal
5—Repeat Item 4	Sternum	ROM of lateral flexion to dominant/unaffected side (degrees)	Frontal
6—Repeat Item 4	Sternum	ROM of lateral flexion to dominant/unaffected side (degrees)	Frontal
7—Lift pelvis from bed at the hemiplegic side	Sacrum	ROM of lateral flexion to dominant/unaffected side (degrees) **	Frontal
8—Repeat Item 7	Sacrum	ROM of lateral flexion to dominant/unaffected side (degrees) **	Frontal
9—Lift pelvis from bed at the unaffected side	Sacrum	ROM of lateral flexion to non-dominant/affected side (degrees) **	Frontal
10—Repeat Item 9	Sacrum	ROM of lateral flexion to non-dominant/affected side (degrees) **	Frontal
**Coordination Subscale**			
1—Rotate upper trunk 6 times	Sternum	Symmetry (%), ROM of average rotation to both side (degrees) and total no. of rotations	Transverse
2—Repeat Item 1 within 6 s	Sternum	Symmetry (%), ROM of average rotation to both side (degrees) and total no. of rotations	Transverse
3—Rotate lower trunk 6 times	Lumbar	Symmetry (%), ROM of average rotation to both side (degrees) and total no. of rotations	Transverse
4—Repeat Item 3 within 6 s	Lumbar	Symmetry (%), ROM of average rotation to both side (degrees) and total no. of rotations	Transverse

* Non-dominant upper limb for healthy participants; affected upper limb for stroke participants. ** In task 7 and 8: The unaffected side is not tested. The stroke participants used lateral flexion towards the unaffected side to be able to lift-up the pelvis on the affected side. As a result, we chose the lateral flexion toward the unaffected (untested) side to be the most important parameter to measure in these tasks. In task 9 and 10: The unaffected side is tested. The stroke participants used lateral flexion towards the affected (untested) side to be able to lift-up the pelvis on the unaffected side. As a result, we chose the lateral flexion toward the affected (untested) side to be the most important parameter to measure in these tasks.

**Table 2:** Participant Characteristics.

Characteristics	Chronic Stroke (N = 20)	Healthy (N = 20)
**Age (years)**	63.2 ± 11.12	62.75 ± 11.67
	Range: 44–79	Range: 41–80
**Gender**		
Male	13	10
Female	7	10
**Hand dominance**		
Right	17	16
Left	3	4
**Affected upper limb**		
Right	5	N/A
Left	15	
**Trunk Impairment Scale (TIS)**	15.65 ± 2.70	22.8 ± 0.62
	Range: 10–23	Range: 2–23
**Number of participants with TIS:**		
≤10 (poor trunk control)	1	0
11–19 (fair trunk control)	18	0
≥20 (good trunk control)	1	20

**Table 3:** Spearman rank correlation of iTIS data with cTIS-V2 score (dynamic subscale) in the stroke group.

cTIS-V2 Tasks	Parameter of Interest	cTIS-V2 Score = 0 Mean ± SD	cTIS-V2 Score = 1 Mean ± SD	r-Value	Sig.
**Dynamic Sitting Balance Subscale**					
1, 2—Touch the bed with the hemiplegic elbow	ROM of lateral flexion to affected side (degrees)	29.21 ± 5.18	21.70 ± 7.18	−0.59 **	0.006
3—Touch the bed with the hemiplegic elbow without compensation	ROM of lateral flexion to affected side (degrees)	32.41 ± 10.36	25.21 ± 3.61	−0.26	0.45
4, 5—Touch the bed with the unaffected elbow	ROM of lateral flexion to unaffected side (degrees)	35.82 ± 4.98	29.75 ± 5.95	−0.52 *	0.02
6—Touch the bed with the unaffected elbow without compensation	ROM of lateral flexion to unaffected side (degrees)	36.31 ± 8.59	32.41 ± 5.30	−0.24	0.41
7—Lift pelvis from bed at the hemiplegic side	ROM of lateral flexion to unaffected side (degrees)	13.74 ± 3.5	21.46 ± 4.9	0.52 *	0.01
8—Lift pelvis from bed at the hemiplegic side without compensation	ROM of lateral flexion to affected side (degrees)	18.92 ± 6.96	21.04 ± 4.65	0.23	0.33
9—Lift pelvis from bed at the unaffected side	ROM of lateral flexion to unaffected side (degrees)	15.29 ± 2.60	18.42 ± 3.24	0.47 *	0.03
10—Lift pelvis from bed at the unaffected side without compensation	ROM of lateral flexion to unaffected side (degrees)	17.81 ± 2.20	17.49 ± 4.04	−0.10	0.67

r value = correlation coefficient; Sig. = significance level. ** Correlation is significant at ≤0.01. * Correlation is significant at ≤0.05.

**Table 4:** Spearman rank correlation of iTIS data with cTIS-V2 score (coordination subscale) in stroke group.

cTIS-V2 Tasks	Parameter of Interest	cTIS-V2 Score = 0 Mean ± SD	cTIS-V2 Score = 1 Mean ± SD	cTIS-V2 Score = 2 Mean ± SD	r-Value	Sig.
Coordination Subscale						
1—Rotate upper trunk 6 times	Symmetry (%)	84.86 ± 14.40	78.46 ± 18.9	94.51 ± 3.61	0.54 *	0.02
	ROM of average rotation to affected side (degrees)	17.76 ± 9.00	21.45 ± 9.43	28.01 ± 4.21	0.57 **	0.01
	ROM of average rotation to unaffected side (degrees)	26.54 ± 19.73	15.65 ± 7.80	25.49 ± 4.20	0.32	0.17
	Total number of rotations	5 ± 0	6 ± 0	6 ± 0	0.59 **	0.007
2—Repeat Item 1 within 6 s	Symmetry (%)	78.94 ± 18.24	97.61 ± 1.29		0.71 **†	0.001
	ROM of average rotation to affected side (degrees)	20.71 ± 12.04	26.53 ± 4.72		0.34	0.14
	ROM of average rotation to unaffected side (degrees)	18.67 ± 11.91	26.07 ± 3.79		0.32	0.17
	Total number of rotations	5.15 ± 0.68	6 ± 0		0.64 **†	0.002
3—Rotate lower trunk 6 times	Symmetry (%)	69.42 ± 16.52	74.36 ± 20.57	85.89 ± 8.80	0.28	0.24
	ROM of average rotation to affected side (degrees)	9.22 ± 6.75	9.01 ± 4.48	7.46 ± 3.50	−0.03	0.87
	ROM of average rotation to unaffected side (degrees)	7.20 ± 4.19	8.51 ± 4.29	6.87 ± 0.45	0.02	0.92
	Total number of rotations	6 ± 0	6 ± 0	5.20 ± 0.45	0.74 ** †	0.001
4—Repeat Item 3 within 6 s	Symmetry (%)	77.20 ± 18.98	88.94 ± 11.02		0.37	0.11
	ROM of average rotation to affected side (degrees)	9.18 ± 4.98	8.08 ± 4.88		−0.14	0.55
	ROM of average rotation to unaffected side (degrees)	7.39 ± 4.48	9.21 ± 4.66		0.20	0.39
	Total number of rotations	5.53 ± 0.51	6 ± 0		0.46 *	0.04

r value= correlation coefficient; Sig. = significance level. ** Correlation is significant at ≤0.01. * Correlation is significant at ≤0.05. † Correlation is significant at ≤0.006.

**Table 5:** Differences in iTIS parameters between stroke and healthy participants.

cTIS-V2 Tasks	Parameter of Interest	Stroke Mean ± SD	Healthy Mean ± SD	Mean Diff ± SD	95% CI for Mean Diff	*p*-Value
**Dynamic Sitting Balance Subscale**						
Task 1	ROM of lateral flexion to non-dominant/affected side (degrees)	24.66 ± 7.75	37.23 ± 5.70	−12.57 ± 7.49	−16.93 to −8.21	0.000 *
Task 2	ROM of lateral flexion to non-dominant/affected side (degrees)	21.51 ± 11.22	34 ± 10.89	12.49 ± 13.95	−21.21 to −3.75	0.007 *
Task 3	ROM of lateral flexion to non-dominant/affected side (degrees)	28.78 ± 7.7	35.39 ± 8.09	6.61 ± 10.49	−13.18 to −0.037	0.05 *
Task 4	ROM of lateral flexion to dominant/unaffected side (degrees)	34.04 ± 6.62	35.04 ± 7.09	−1 ± 12.8	−5.57 to 3.57	0.66
Task 5	ROM of lateral flexion to dominant/unaffected side (degrees)	33.21 ± 9.55	34.37 ± 8.26	−1.16 ± 14.25	−7.40 to 5.10	0.71
Task 6	ROM of lateral flexion to dominant/unaffected side (degrees)	27.01 ± 9.23	34.87 ± 8.43	7.86 ± 12.71	−14.08 to −1.63	0.01 *
Task 7	ROM of lateral flexion to dominant/unaffected side (degrees)	20.11 ± 5.54	22.44 ± 5.2	−2.32 ± 8.94	−5.97 to 1.31	0.20
Task 8	ROM of lateral flexion to dominant/unaffected side (degrees)	19.02 ± 5.51	23.29 ± 6.99	−4.27 ± 9.97	−8.46 to −0.082	0.05 *
Task 9	ROM of lateral flexion to non-dominant/affected side (degrees)	16.38 ± 4.41	23.74 ± 6.18	−7.36 ± 7.25	−10.86 to −3.86	0.000 *
Task 10	ROM of lateral flexion to non-dominant/affected side (degrees)	16.50 ± 5.02	25.93 ± 6.54	−9.42 ± 8.31	−13.45 to −5.39	0.000 *
**Coordination Subscale**						
Task 1	Symmetry (%)	87.58 ± 13.15	93.75 ± 7.92	−6.17 ± 17.30	−14.08 to 1.74	0.12
	ROM of average rotation to non-dominant/affected side (degrees)	22.17 ± 6.32	16.86 ± 7.19	5.31 ± 11.06	0.32 to 10.30	0.04 *
	ROM of average rotation to dominant/unaffected side (degrees)	20.92 ± 8.70	30.68 ± 6.86	−9.76 ± 10.83	−15.50 to −4.01	0.002 *
	Total number of rotations	5.93 ± 0.25	6 ± 0.00	−0.06 ± 0.31	−0.19 to 0.06	0.310
Task 2	Symmetry (%)	86.34 ± 13.16	91.72 ± 5.90	−5.37 ± 13.27	−12.41 to 1.66	0.129
	ROM of average rotation to non-dominant/affected side (degrees)	32.59 ± 7.18	22.70 ± 8.69	9.88 ± 10.74	4.15 to 15.62	0.001 *
	ROM of average rotation to dominant/unaffected side (degrees)	21.85 ± 8.28	29.15 ± 7.91	−7.30 ± 10.53	−13.06 to −1.54	0.015 *
	Total number of rotations	5.60 ± 0.73	6 ± 0.00	−0.40 ± 0.76	−0.75 to −0.046	0.03 *
Task 3	Symmetry (%)	78.42 ± 17.92	87.16 ± 9.84	−8.74 ± 25.58	−19.36 to 1.87	0.10
	ROM of average rotation to non-dominant/affected side (degrees)	7.43 ± 3.83	14.95 ± 7.52	−7.51 ± 9.49	−12.37 to −2.64	0.004 *
	ROM of average rotation to dominant/unaffected side (degrees)	7.89 ± 2.73	13.11 ± 5.55	−5.21 ± 5.95	−8.79 to −1.64	0.006 *
	Total number of rotations	5.83 ± 0.38	6 ± 0.00	−0.16 ± 0.30	−0.35 to 0.02	0.08
Task 4	Symmetry (%)	80.89 ± 18.03	87 ± 11.34	−6.11 ± 21.50	−16.98 to 4.76	0.26
	ROM of average rotation to non-dominant/affected side (degrees)	9.71 ± 4.78	15.38 ± 5.04	−5.67 ± 9.51	−9.22 to −2.12	0.003 *
	ROM of average rotation to dominant/unaffected side (degrees)	8.57 ± 4.60	13.27 ± 4.61	−4.69 ± 7.21	−8.03 to −1.36	0.007 *
	Total number of rotations	5.75 ± 0.44	6 ± 0.00	−0.25 ± 0.74	−0.47 to −0.021	0.033 *

* *p* ≤ 0.05.

**Table 6:** Differences in iTIS parameters between stroke participants scoring zero, one and two on the clinical TIS.

TIS-V2 Tasks	Parameter of Interest	cTIS Score = 0 Mean ± SD	cTIS Score = 1 Mean ± SD	Mean Diff ± SD	95% CI for Mean Diff	*p*-Value
Dynamic Sitting Balance Subscale						
1, 2—Touch the bed or table with the hemiplegic elbow	ROM of lateral flexion to affected side (degrees)	29.21 ± 5.18	21.70 ± 7.18	7.51 ± 2.80	1.62 to 13.39	0.01 *
3—Touch the bed or table with the hemiplegic elbow without compensation	ROM of lateral flexion to affected side (degrees)	32.41 ± 10.36	25.21 ± 3.61	7.19 ± 6.31	−7.36 to 21.76	0.28
4, 5—Touch the bed or table with the unaffected elbow	ROM of lateral flexion to unaffected side (degrees)	35.82 ± 4.98	29.75 ± 5.95	6.07 ± 2.65	0.42 to 11.72	0.03 *
6—Touch the bed or table with the unaffected elbow without compensation	ROM of lateral flexion to unaffected side (degrees)	36.31 ± 8.59	32.41 ± 5.30	3.89 ± 3.89	−4.67 to 12.46	0.33
7—Lift pelvis from bed or table at the hemiplegic side	ROM of lateral flexion to affected side (degrees)	13.74 ± 3.5	21.46 ± 4.9	−7.71 ± 3.02	−14.07 to −1.36	0.02 *
8—Lift pelvis from bed or table at the hemiplegic side without compensation	ROM of lateral flexion to affected side (degrees)	18.92 ± 6.96	21.04 ± 4.65	−2.12 ± 2.59	−7.57 to 3.32	0.42
9—Lift pelvis from bed or table at the unaffected side	ROM of lateral flexion to unaffected side (degrees)	15.29 ± 2.60	18.42 ± 3.24	−3.12 ± 1.44	−6.17 to −0.07	0.04 *
10—Lift pelvis from bed or table at the unaffected side without compensation	ROM of lateral flexion to unaffected side (degrees)	17.81 ± 2.20	17.49 ± 4.04	0.32 ± 1.93	−3.76 to 4.41	0.86
**Coordination Subscale**						
**Task 2 & 4 (Independent Samples *t*-Test)**						
2—Repeat Item 1 within 6 s	Symmetry (%)	78.94 ± 18.24	97.61 ± 1.29	−18.66 ± 6.99	−33.35 to −3.97	0.01 *
	ROM of average rotation to non-dominant/affected side (degrees)	20.71 ± 12.04	26.53 ± 4.72	−5.81 ± 4.78	−15.86 to 4.23	0.24
	ROM of average rotation to dominant/unaffected side (degrees)	18.67 ± 11.91	26.07 ± 3.79	−7.40 ± 4.67	−17.22 to 2.41	0.13
	Total number of rotations	5.15 ± 0.68	6 ± 0	−0.84 ± 0.26	−1.40 to −0.29	0.001 *
4—Repeat Item 3 within 6 s	Symmetry (%)	77.20 ± 18.98	88.94 ± 11.02	−11.74 ± 8.40	−29.47 to 5.99	0.18
	ROM of average rotation to non-dominant/affected side (degrees)	9.18 ± 4.98	8.08 ± 4.88	1.09 ± 2.44	−4.06 to 6.25	0.65
	ROM of average rotation to dominant/unaffected side (degrees)	7.39 ± 4.48	9.21 ± 4.66	−1.82 ± 2.23	−6.54 to 2.90	0.42
	Total number of rotations	5.53 ± 0.51	6 ± 0	−0.46 ± 0.21	−0.91 to −0.007	0.04 *
**Task 1 & 3 (One-Way ANOVA Test)**						
**cTIS-V2 Tasks**	**Parameter of Interest**	**cTIS Score = 0** **Mean ± SD**	**cTIS Score = 1** **Mean ± SD**	**cTIS Score = 2** **Mean ± SD**		***p*-Value**
1—Rotate upper trunk 6 times	Symmetry (%)	84.86 ± 14.40	78.46 ± 18.99	94.51 ± 3.61		0.04 *
	ROM of average rotation to non-dominant/affected side (degrees)	17.76 ± 9.00	21.45 ± 9.43	28.01 ± 4.21		0.31
	ROM of average rotation to dominant/unaffected side (degrees)	26.54 ± 19.73	15.65 ± 7.80	25.49 ± 4.20		0.002 *
	Total number of rotations	5 ± 0	6 ± 0	6 ± 0		0.001
3—Rotate lower trunk 6 times	Symmetry (%)	69.42 ± 16.52	74.36 ± 20.57	85.89 ± 8.80		0.48
	ROM of average rotation to non-dominant/affected side (degrees)	9.22 ± 6.75	9.01 ± 4.48	7.46 ± 3.50		0.87
	ROM of average rotation to dominant/unaffected side (degrees)	7.20 ± 4.19	8.51 ± 4.29	6.87 ± 0.45		0.74
	Total number of rotations	5.20 ± 0.45	6 ± 0	6 ± 0		0.001

* *p* ≤ 0.05. ** *p* ≤ 0.01.

**Table 7:** Discriminant ability of iTIS parameters in distinguishing between stroke participants with impairment (scored zero on cTIS-V2 tasks) and those without trunk impairment (scored one, or two on cTIS-V2 tasks).

TIS-V2 Tasks	iTIS Parameter	AUC	Std. Error	Sig.	95% CI	Cut-Off Point (Degrees) to be Scored Zero in cTIS	Sensitivity	Specificity
**Dynamic Sitting Balance Subscale**								
1, 2—Touch the bed or table with the hemiplegic elbow	ROM of lateral flexion to affected side (degrees)	0.84	0.09	0.01	0.64–1	≥23.5	0.90	0.80
3—Touch the bed or table with the hemiplegic elbow without compensation	ROM of lateral flexion to affected side (degrees)	0.67	0.17	0.42	0.32–1	≥26.8	0.71	0.67
4, 5—Touch the bed or table with the unaffected elbow	ROM of lateral flexion to unaffected side (degrees)	0.80	0.12	0.03	0.57–1	≥30.7	0.88	0.75
6—Touch the bed or table with the unaffected elbow without compensation	ROM of lateral flexion to unaffected side (degrees)	0.64	0.17	0.39	0.30–98	≥33.8	0.66	0.58
7—Lift pelvis from bed or table at the hemiplegic side	ROM of lateral flexion to affected side (degrees)	0.92	0.06	0.02	0.79–1	≤15.8	1	0.88
8—Lift pelvis from bed or table at the hemiplegic side without compensation	ROM of lateral flexion to affected side (degrees)	0.63	0.15	0.32	0.33–0.94	≤23.2	0.71	0.46
9—Lift pelvis from bed or table at the unaffected side	ROM of lateral flexion to unaffected side (degrees)	0.78	0.11	0.04	0.56–1	≤16.5	0.85	0.75
10—Lift pelvis from bed or table at the unaffected side without compensation	ROM of lateral flexion to unaffected side (degrees)	0.43	0.14	0.65	0.14–0.71	≤18.9	0.80	0.30
**Coordination Subscale**								
1—Rotate upper trunk 6 times	Symmetry (%)	0.87	0.08	0.01	0.70–1	90	0.85	0.66
	ROM of average rotation to non-dominant/affected side (degrees)	0.85	0.09	0.01	0.66–1	24.5	0.85	0.75
	ROM of average rotation to dominant/unaffected side (degrees)	0.77	0.11	0.05	0.56–0.99	23.4	0.71	0.75
	Total number of rotations	0.58	0.13	0.55	0.32–0.85	5.5	1	0.17
2—Repeat Item 1 within 6 s	Symmetry (%)	0.93	0.05	0.002	0.82–1	96.7	0.85	0.84
	ROM of average rotation to non-dominant/affected side (degrees)	0.70	0.11	0.14	0.47–0.93	24.7	0.71	0.69
	ROM of average rotation to dominant/unaffected side (degrees)	0.70	0.12	0.16	0.45–0.93	23.11	0.85	0.61
	Total number of rotations	0.84	0.08	0.01	0.67–1	5.5	1	0.69
3—Rotate lower trunk 6 times	Symmetry (%)	0.70	0.14	0.31	0.41–0.96	76.6	1	0.56
	ROM of average rotation to non-dominant/affected side (degrees)	0.40	0.17	0.58	0.05–0.74	5.8	0.66	0.31
	ROM of average rotation to dominant/unaffected side (degrees)	0.42	0.12	0.65	0.18–0.66	6.1	1	0.37
	Total number of rotations	0.63	0.16	0.50	0.32–0.93	5.5	1	0.25
4—Repeat Item 3 within 6 s	Symmetry (%)	0.73	0.12	0.11	0.48–0.98	80.8	0.83	0.69
	ROM of average rotation to non-dominant/affected side (degrees)	0.41	0.14	0.53	0.12–0.69	6.9	0.66	0.38
	ROM of average rotation to dominant/unaffected side (degrees)	0.62	0.14	0.38	0.34–0.91	7	0.83	0.61
	Total number of rotations	0.73	0.11	0.11	0.50–0.95	5.5	1	0.46

AUC = area under curve; CI = confidence interval.

## Supplementary Materials

The following are available online at https://www.mdpi.com/1424-8220/20/6/1699/s1.
